# Replication Stress and Chromatin Context Link ATM Activation to a Role in DNA Replication

**DOI:** 10.1016/j.molcel.2013.10.019

**Published:** 2013-12-12

**Authors:** Monica M. Olcina, Iosifina P. Foskolou, Selvakumar Anbalagan, Joana M. Senra, Isabel M. Pires, Yanyan Jiang, Anderson J. Ryan, Ester M. Hammond

**Affiliations:** 1 The Gray Institute for Radiation Oncology and Biology, Department of Oncology, University of Oxford, Oxford, OX3 7DQ, UK

## Abstract

ATM-mediated signaling in response to DNA damage is a barrier to tumorigenesis. Here we asked whether replication stress could also contribute to ATM signaling. We demonstrate that, in the absence of DNA damage, ATM responds to replication stress in a hypoxia-induced heterochromatin-like context. In certain hypoxic conditions, replication stress occurs in the absence of detectable DNA damage. Hypoxia also induces H3K9me3, a histone modification associated with gene repression and heterochromatin. Hypoxia-induced replication stress together with increased H3K9me3 leads to ATM activation. Importantly, ATM prevents the accumulation of DNA damage in hypoxia. Most significantly, we describe a stress-specific role for ATM in maintaining DNA replication rates in a background of increased H3K9me3. Furthermore, the ATM-mediated response to oncogene-induced replication stress is enhanced in hypoxic conditions. Together, these data indicate that hypoxia plays a critical role in the activation of the DNA damage response, therefore contributing to this barrier to tumorigenesis.

## Highlights

• In the absence of damage, ATM responds to replication stress in H3K9me3-rich contexts
• The levels of ATM-S1981, H3K9me3, and hypoxia correlate well in vivo
• ATM maintains DNA replication rates in hypoxia
• ATM prevents the accumulation of DNA damage in hypoxic conditions

## Introduction

Replication stress (RS) leads to fork stalling as a result of nucleotide pool depletion and/or the generation of DNA lesions (Murga et al., 2011). RS triggers a DNA damage response (DDR) in an attempt to resolve the insult, ensure fork integrity, and restart DNA synthesis (Harper and Elledge, 2007). The DDR is a complicated signaling cascade composed of sensors, transducers, and effectors that collectively orchestrate a response to the initial stress: RS or DNA single- or double-strand breaks (DSBs). The ataxia-telangiectasia mutated (ATM) serine/threonine kinase is a phosphatidyl inositol-3-kinase-like kinase that has been characterized as an important component of the DDR involved in sensing and responding to DSBs (Jackson and Bartek, 2009). ATM is present as an inactive dimer that becomes activated upon dissociation into monomers and intermolecular autophosphorylation on serine 1981 (Bakkenist and Kastan, 2003). Phosphatases such as PP2A and WIP-1 are also important for the regulation of overall ATM activity following DNA damage (Goodarzi et al., 2004; Shreeram et al., 2006).

Low oxygen levels (hypoxia) occur in the majority of solid tumors and are associated with tumor development and progression. Importantly, severe hypoxia (<0.1% O_2_) can induce RS and activation of the DDR (Bencokova et al., 2009; Bristow and Hill, 2008). In hypoxia, areas of single-stranded DNA accumulate, leading to ataxia-telangiectasia and Rad3-related (ATR) activation. Surprisingly, the hypoxia-induced DDR also includes rapid activation of ATM, despite the lack of associated DNA damage. It is important to note that the activity of the MRN (Mre11-Rad50-Nbs1) complex is not necessary for the activation of ATM under hypoxic conditions. Interestingly, oxidative stresses such as those attributable to H_2_O_2_ also induce ATM activity independent of the MRN complex in the absence of DNA breaks (Guo et al., 2010a). However, ATM signaling under oxidative stress differs from hypoxia-induced ATM, because the former does not include KAP-1 (S824) or H2AX (S139) phosphorylation (Bencokova et al., 2009; Guo et al., 2010b). KAP-1 phosphorylation at S824 occurs in an ATM-dependent manner in response to a variety of stresses including ionizing radiation (IR) and hypoxia (Ziv et al., 2006).

Here we have verified that ATM can respond to RS in the absence of DNA damage, providing this occurs in the presence of increased levels of H3K9me3. This observation led us to question the role of ATM in these conditions. Most significantly, loss of ATM during exposure to hypoxia-induced RS led to an accumulation of DNA damage and a decrease in DNA replication rates. This study highlights a stress- and context-specific role for ATM in DNA replication in conditions of RS.

## Results

### Replication Stress Can Induce ATM Activity in the Absence of DNA Damage

**Figure 1:**
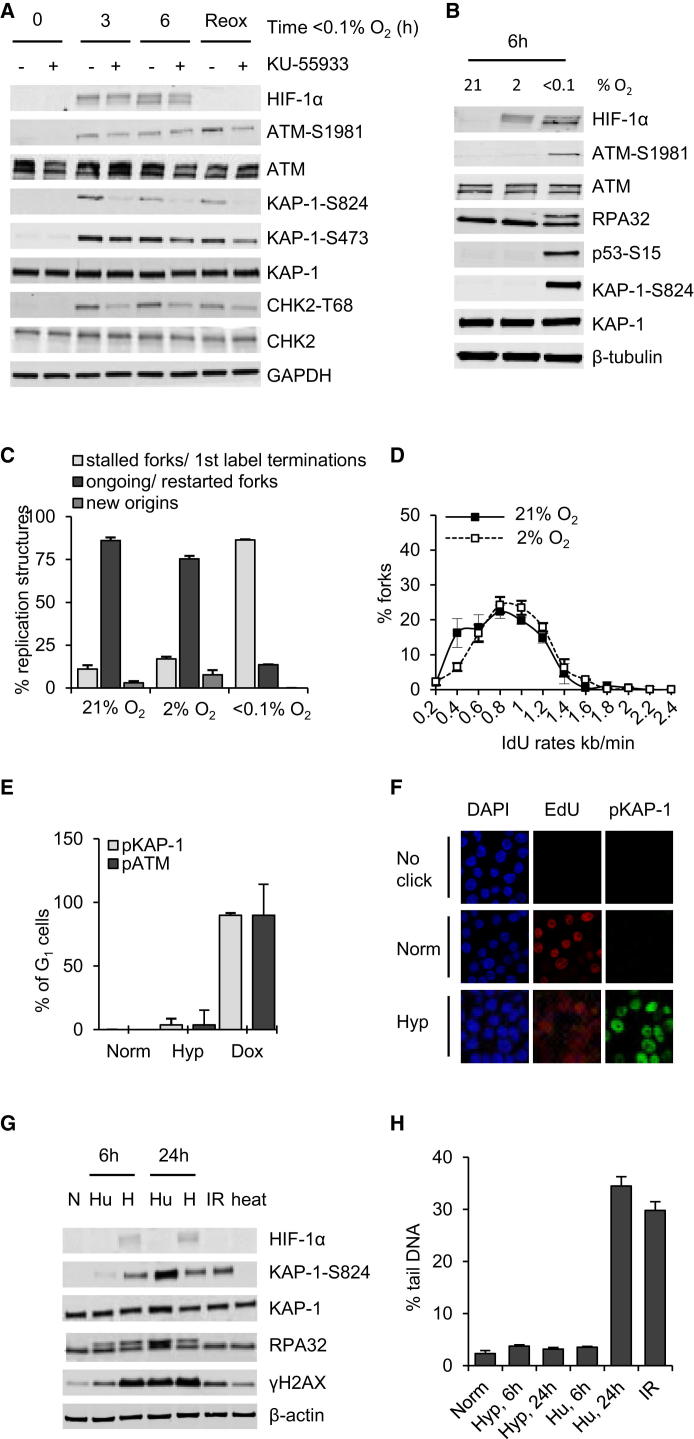
Hypoxia-Induced Replication Stress and ATM Activity (A) RKO cells were exposed to <0.1% O_2_ for the times indicated in the presence of DMSO or ATM inhibitor (KU-55933). Reox (6 hr, <0.1% O_2_ followed by 1 hr of 21% O_2_). Western blotting (WB) was carried out with the antibodies indicated; GAPDH was the loading control. (B) RKO cells were exposed to 21%, 2%, or <0.1% O_2_. WB was carried out as indicated; β-tubulin was the loading control. (C) RKO cells were exposed to 21%, 2%, or <0.1% O_2_ and DNA fibers were scored. Shown is a graph of the replication structures. (D) The graph indicates the distribution of fork rates for the second label for the forks scored in (C) for cells exposed to normoxia (21% O_2_) or hypoxia (2% O_2_, 6 hr). IdU, 5-iodo-2′-deoxyuridine. (E) The quantification of the number of stained G_1_ cells is shown. pKAP-1, KAP-1-S824; pATM, ATM-S1981. Norm (21% O_2_); Hyp (<0.1% O_2_, 6 hr); Doxorubicin (Dox; 4 hr). (F) RKO cells were labeled with EdU and costained with KAP-1-S824. Hyp (<0.1% O_2_, 6 hr). A representative image of the colocalization of KAP-1-S824 and EdU staining is shown. DAPI, 4′,6-diamidino-2-phenylindole. (G) RKO cells were exposed to N (21% O_2_), Hu (6 or 24 hr), H (<0.1% O_2_, 6 or 24 hr), IR (5 Gy, harvested 30 min post IR), or heat (42.5°C, 1 hr). WB was carried out with the antibodies indicated; β-actin was the loading control. (H) RKO cells were treated as shown and an alkaline comet assay was carried out. Norm (21% O_2_), Hyp (<0.1% O_2_, 6 or 24 hr), Hu (6 or 24 hr), IR (5 Gy, lysed immediately after IR). Shown is the % comet tail DNA. Data are represented as mean ± SEM. See also Figure S1.

Exposure to hypoxia (<0.1% O_2_) does not induce detectable levels of DNA damage using available assays (Bencokova et al., 2009) (Figures S1A–S1C available online). Despite this, ATM is rapidly phosphorylated and downstream targets are phosphorylated in an ATM-dependent manner. Hypoxia-induced ATM targets include both CHK2 (T68) and KAP-1 (S824) (Figure 1A). An increase in phosphorylation of KAP-1 at residue 473 was also observed in response to hypoxia, but was not significantly affected by ATM inhibition, in agreement with the finding that it is dependent on CHK1 (Blasius et al., 2011). To study a possible link between the hypoxia-induced ATM response and RS, we investigated the activation of ATM in response to 2% and <0.1% O_2_. Both of these levels of oxygen induce a hypoxic response, including HIF-1α stabilization. However, only in conditions of <0.1% O_2_ is ATM phosphorylated at serine 1981 and downstream ATM/ATR targets activated (Figure 1B). Importantly, exposure to <0.1% O_2_ leads to phosphorylation of RPA32, which is again indicative of RS in these conditions. Analysis of replication structures in 21%, 2%, and <0.1% O_2_ demonstrated that hypoxia (<0.1% O_2_) induces a significant increase in the number of stalled forks (Pires et al., 2010). In contrast, the numbers of stalled or ongoing forks exposed to 21% or 2% O_2_ are similar (Figure 1C). In addition, replication rates are unaffected by exposure to 2% O_2_ when compared to the replication rates of cells exposed to 21% O_2_ (Figure 1D). These data suggest that hypoxia-induced RS signals to ATM and, therefore, that in hypoxic conditions, <0.1% O_2_, ATM-mediated signaling should be restricted to S phase. We determined that KAP-1 is phosphorylated at S824 in approximately 50% of RKO cells exposed to <0.1% O_2_ (Figure S1D). In contrast, a population enriched for G_1_ cells and exposed to hypoxia showed little or no induction of KAP-1-S824 or ATM-S1981 (Figure 1E). By labeling cells with 5-ethynyl-2′-deoxyuridine (EdU), we confirmed that, in hypoxia, cells in S phase accumulated KAP-1-S824 (Figure 1F; Figure S1E). This was further supported by fluorescence-activated cell sorting analysis (Figure S1F).

The ATM-dependent signaling to KAP-1 in response to RS induced by hypoxia was surprising given that previous studies have shown that KAP-1 is only phosphorylated in the presence of DNA damage (Goodarzi et al., 2009). RKO cells were exposed to hydroxyurea (Hu) or hypoxia (<0.1% O_2_) for either 6 or 24 hr. Irradiation was used as a DNA-damaging control, and heat (42°C) as a non-DNA-damaging control (Figure S1G) (Hunt et al., 2007). Acute exposure (6 hr) to both Hu and hypoxia led to phosphorylation of RPA32, confirming RS (Figure 1G). Importantly, although the short acute exposure to both Hu and hypoxia induced RS, neither induced DNA damage detectable by comet assay (Figure 1H). In contrast to the response to hypoxia-induced RS, acute exposure to Hu did not induce KAP-1 phosphorylation. After a longer exposure to Hu (24 hr) significant KAP-1 phosphorylation was observed, although this correlated with an induction of DNA damage detected by comet assay (Figure 1H). These data suggest that ATM does respond to RS but that this is context specific. Interestingly, loss of ATM does not sensitize cells to replication stresses such as Hu (Bolderson et al., 2004). However, inhibition of ATM, using KU-55933, during exposure to hypoxia (<0.1% O_2_) led to decreased colony survival, suggesting an important role for ATM in these conditions (Figure S1H).

### Hypoxia-Induced H3K9me3 Correlates with ATM Activity In Vitro and In Vivo

**Figure 2:**
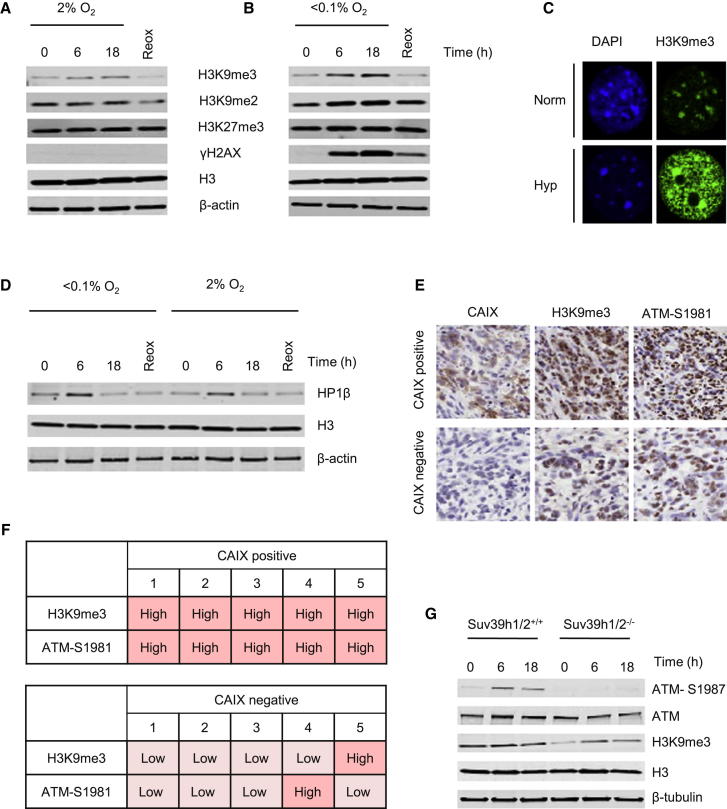
Hypoxia-Induced H3K9me3 Correlates with ATM Activity In Vitro and In Vivo (A and B) RKO cells were treated with either 2% (A) or <0.1% O_2_ (B). Reox (6 hr, <0.1% O_2_ followed by 2 hr of 21% O_2_). The chromatin modifications investigated are shown between the Western blots. (C) Mouse NIH 3T3 cells were fixed and stained for H3K9me3 following exposure to Norm (21% O_2_) or Hyp (<0.1% O_2_, 20 hr). (D) RKO cells were treated as in (A) and (B). WB was carried out. (E) Serial sections from Calu6 xenografts were stained for H3K9me3, CAIX, or ATM-S1981. Shown are representative images of regions positive or negative for CAIX staining and the corresponding H3K9me3 and ATM-S1981 staining in brown. (F) Five CAIX-positive and -negative regions were scored for both H3K9me3 and ATM-S1981. (G) Suv39h1/2^+/+^ and Suv39h1/2^−/−^ MEFs were treated as indicated (Hyp, <0.1% O_2_). WB was carried out; β-tubulin was the loading control. See also Figure S2.

Hypoxia-induced RS occurs within a specific chromatin context. Many hypoxia-induced histone modifications have been reported (Johnson et al., 2008). However, the consequences of hypoxia-induced chromatin changes as well as the regulation of many of the enzymes that lead to such changes are poorly understood (Melvin and Rocha, 2012). We investigated the induction of a number of histone marks in hypoxia and after reoxygenation. As expected, hypoxia (<0.1% O_2_) led to the induction of pan-nuclear γH2AX indicative of RS, whereas milder hypoxic conditions (2% O_2_) did not (Figures 2A and 2B; Figure S1A) (Toledo et al., 2011). An increase in H3K9me3 under both hypoxic conditions was observed, which returned to baseline levels after reoxygenation. In contrast, we only saw a mild induction in H3K9me2 in response to <0.1% O_2_, and did not observe marked differences in H3K27me3 levels in response to either <0.1% or 2% O_2_. Interestingly, the increase in H3K9me3 was inversely related to the level of oxygen. H3K9me3 is enriched in chromocenters corresponding to centrometic and pericentric heterochromatin (Guenatri et al., 2004). In response to hypoxia (<0.1% O_2_), H3K9me3 enrichment occurred throughout the nucleus and in all cell-cycle phases (Figure 2C; Figure S2A). H3K9me3 recruits chromodomain-containing proteins such as HP1β, which can regulate heterochromatin formation (Nielsen et al., 2002). HP1β-H3K9me3 binding is proposed to allow H3K9 methylation on neighboring nucleosomes through the recruitment of Suv39h1 and other methyltransferases by HP1β (Loyola et al., 2009). The expression of HP1β in hypoxia, however, has not been studied to date. Here we observed a transient increase in expression of chromatin-bound HP1β after 6 hr of hypoxia (Figure 2D; Figure S2B).

We observed that two methyltransferases responsible for H3K9me3, SETDB1 and Suv39h2, were induced at the protein but not mRNA level in response to hypoxia (<0.1% O_2_) (Figures S2C–S2E). Furthermore, the protein levels of SETDB1 were decreased in response to reoxygenation, reflecting the drop in H3K9me3 observed in response to reoxygenation. Levels of Suv39h2 were notably increased in response to reoxygenation and IR, suggesting that this methyltransferase is DNA damage responsive.

We hypothesized that RS in the presence of increased H3K9me3 could be linked to ATM activation in the absence of damage. In order to verify that H3K9me3 occurs in the hypoxic areas of tumors and correlates with ATM activation, subcutaneous Calu6 xenograft tumors were stained for H3K9me3, the hypoxia marker CAIX, and ATM-S1981. Importantly, in CAIX-positive regions of the tumor, there was a clear association with both high expression of H3K9me3 and ATM-S1981 (Figures 2E and 2F; Figure S2F).

### Hypoxia-Induced H3K9me3 Is Required for ATM Activity

To test whether increased H3K9me3 was required for ATM signaling in hypoxia, we investigated the effects of loss of H3K9me3 on ATM activation. One of the roles of KAP-1 is to act as a transcriptional corepressor by facilitating H3K9me3 spreading (Schultz et al., 2002). Upon treatment with KAP-1 siRNA a modest reduction in H3K9me3 was observed. Interestingly, this correlated with a similar reduction in phosphorylated ATM (Figures S2G and S2H). We then used Suv39h1/2^+/+^ and Suv39h1/2^−/−^ mouse embryonic fibroblasts (MEFs) to investigate the direct role of these two methyltransferases in ATM phosphorylation in hypoxia. As expected, the Suv39h1/2^−/−^ MEFs showed a decreased level of H3K9me3 in both normoxia and hypoxia. Most importantly, ATM phosphorylation was markedly decreased in these cells following exposure to hypoxia (<0.1% O_2_) (Figure 2G; Figure S2I). As previously reported, we also observed reduced ATM phosphorylation in response to IR in the Suv39h1/2^−/−^ cell line (Figure S2J) (Sun et al., 2009). Lentiviral knockdown of Suv39h1 and Suv39h2 also showed a reduction in H3K9me3 levels and impaired hypoxia-induced ATM activity (Figure S2K). ATM activation in response to RS, therefore, occurs in a specific hypoxia-induced chromatin context, which includes increased levels of H3K9me3.

Because H3K9me3 is associated with gene repression, we hypothesized that this chromatin mark may be contributing to hypoxia-induced ATM activation through repression of a factor that normally inhibits ATM phosphorylation, such as an ATM-specific phosphatase. In support of this, both WIP-1 and PP2A-C appear genetically regulated by Suv39h1/2 (Figures S2L and S2M). We measured the enrichment of H3K9me3 on PP2A-C by chromatin immunoprecipitation and observed a moderate increase in H3K9me3 enrichment in response to hypoxia (Figures S2N–S2P). In addition, we verified that the chromatin accessibility of the PP2A-C gene was reduced and that mRNA expression decreased in response to hypoxia (Figures S2Q and S2R). Finally, we assessed the functional activity of PP2A-C and found a significant reduction in hypoxic conditions (<0.1% O_2_) (Figure S2S). Collectively, these data suggest that H3K9me3 can facilitate ATM activation in response to hypoxia (<0.1% O_2_) through repression of ATM-specific phosphatases, including PP2A.

### Hypoxia-Induced ATM Has a Role to Play in DNA Replication

**Figure 3:**
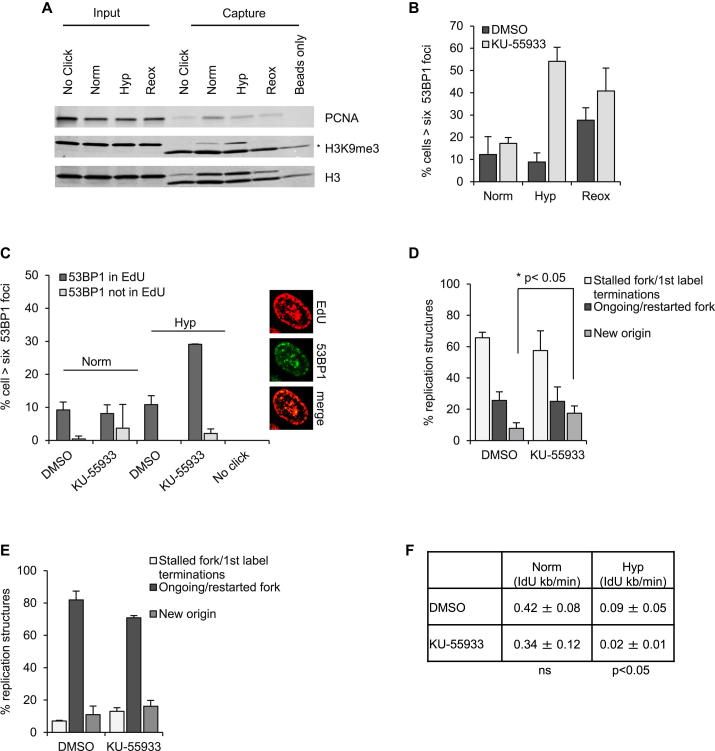
Hypoxia-Induced ATM Has a Role in Replication (A) iPOND was carried out following exposure of cells to either Norm (21% O_2_), Hyp (6 hr, <0.1% O_2_), or Reox (6 hr, <0.1% O_2_ followed by 2 hr of 21% O_2_). The asterisk denotes a nonspecific band observed in all lanes at the end of the gel. (B) RKO cells were treated with DMSO or KU-55933 and exposed to Norm (21% O_2_), Hyp (<0.1% O_2_, 6 hr), or Reox (6 hr, <0.1% O_2_ followed by 1 hr of 21% O_2_). The quantification of 53BP1 foci counted in three independent experiments is shown. (C) RKO cells were labeled with EdU, treated with DMSO or KU-55933, and exposed to Norm (21% O_2_) or Hyp (<0.1% O_2_, 3 hr). EdU labeling times were 1 hr for Norm and 3 hr for Hyp conditions. (D) RKO cells were treated with DMSO or KU-55933 and exposed to hypoxia (<0.1% O_2_, 5 hr) followed by 1 hr of reox (21% O_2_). DNA fibers were scored. (E) RKO cells were treated as above but in normoxia (21% O_2_). (F) The replication rates of cells treated with DMSO or KU-55933 and exposed to Norm (21% O_2_) or Hyp (<0.1% O_2_, 6 hr) were scored. An average of the average IdU incorporation rates for each of three independent experiments is shown. ns, nonsignificance. Data are represented as mean ± SEM. See also Figure S3.

The dependence on H3K9me3 for ATM activation in response to RS in hypoxia led us to hypothesize that ATM could be involved in facilitating replication through H3K9me3-rich regions. To address this possibility, we first investigated the specific localization of H3K9me3 using the recently described iPOND (isolation of proteins on nascent DNA) technique (Sirbu et al., 2012). This technique allowed us to determine whether the hypoxia-induced increase in H3K9me3 was also associated with or around replication forks (Figure S3A). The exposure to EdU was optimized to ensure equal amounts of DNA were pulled down in each condition (Figure S3B). Thymidine-chase experiments were also carried out to ensure specific enrichment of H3K9me3 in areas associated with the replication fork (Figure S3C). iPOND analysis demonstrated that there was an increase in H3K9me3 in the vicinity of the replication fork in hypoxia (<0.1% O_2_). PCNA was used as a replication fork-associated control (Figure 3A). The presence of H3K9me3 and associated heterochromatic factors around the replication fork could contribute to hypoxia-induced RS and potentially lead to a requirement for ATM activity in order to preserve fork integrity. To test this hypothesis, RKO cells were exposed to hypoxia in the presence of the ATM inhibitor and the accumulation of DNA damage was measured by increased 53BP1 focus formation. Whereas no significant increase in damage was seen in KU-55933-treated cells in normoxia, increased DNA damage was observed in hypoxic cells treated with KU-55933 (Figure 3B). Similar results were obtained upon siRNA-mediated depletion of ATM (Figure S3D). Furthermore, ATM inhibition or knockdown following exposure to 2% O_2_ (where no RS occurs) did not lead to a significant increase in DNA damage, suggesting that the role of ATM in preserving fork integrity in hypoxia is restricted to conditions in which RS is observed (Figures S3E and S3F). To investigate this further, H1299 cells expressing Fucci plasmids (Sakaue-Sawano et al., 2008) were used in order to assess the specific cell-cycle phase in which increased 53BP1 foci were observed following exposure to <0.1% O_2_. Notably, the majority of the 53BP1 foci observed in hypoxia following treatment with the ATM inhibitor were present in the S phase population (Figure S3G). To validate this finding, RKO cells were labeled with EdU and exposed to hypoxia in the presence or absence of the ATM inhibitor. The majority of the DNA damage detected was present in the EdU-positive/S phase population (Figure 3C). Interestingly, in cells with EdU staining representative of heterochromatin replication in mid/late S phase, the 53BP1 foci clustered in areas thought to be associated with replicating heterochromatin (Nakayasu and Berezney, 1989). These data support the model that loss of ATM activity leads to the accumulation of DNA damage in hypoxic conditions associated with RS and an H3K9me3-rich environment.

It is important to note that the iPOND analysis also showed a dramatic reduction in H3K9me3 in response to reoxygenation, indicating a dynamic oxygen-dependent regulation of this chromatin modification. Therefore, we predicted that loss of ATM during reoxygenation-induced replication restart would have no effect on the DNA damage levels. In support of this, we saw no increase in reoxygenation-induced DNA damage following ATM inhibition (Figure 3B). These findings suggest that replication should be unaffected by ATM inhibition when H3K9me3 is reduced during reoxygenation-induced replication restart. Notably, DNA fiber analysis showed that in conditions of reoxygenation, replication restart was unaffected by ATM inhibition. Surprisingly, however, following reoxygenation the number of new origins increased in the presence of the ATM inhibitor (Figure 3D; Figure S3H). Importantly, inhibition of ATM under normoxic conditions had a nonsignificant effect on origin firing, pointing to a stress-specific role for ATM in regulating appropriate replication restart in hypoxic conditions (Figure 3E).

The data so far pointed to a role for ATM in facilitating replication in hypoxia where there is an increase in H3K9me3. We measured replication rates in cells in either normoxia or during hypoxia (<0.1% O_2_) following ATM inhibition/knockdown (Figure 3F; Figures S3I and S3J). Loss of ATM did not have a significant effect on replication rates in normoxia. In contrast, replication rates were significantly slower in hypoxic cells treated with the ATM inhibitor compared to the hypoxic controls. These data demonstrate a stress-specific role for ATM in DNA replication.

### Replication Stress in Hypoxic Conditions Leads to ATM Activation

**Figure 4:**
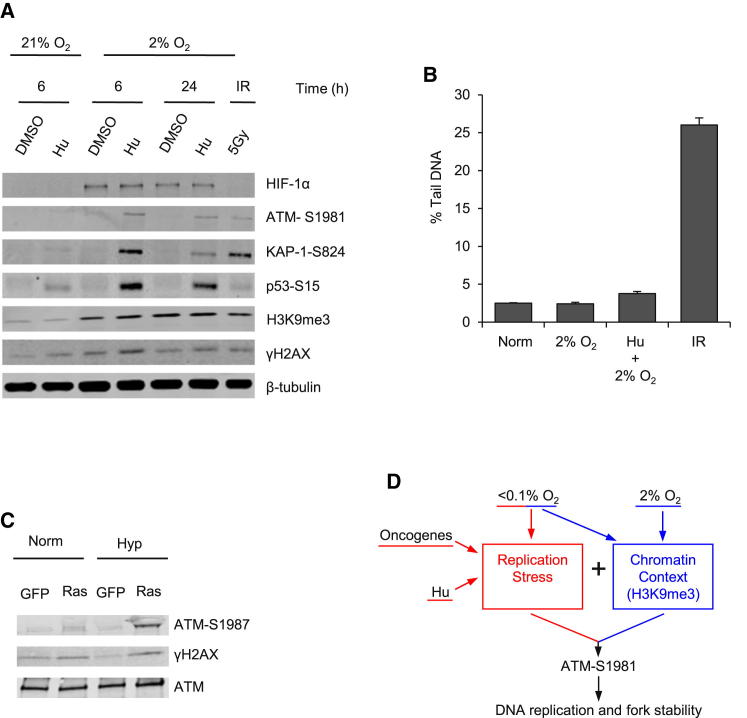
Both Hu- and Oncogene-Induced Replication Stress Lead to ATM Activation in Hypoxic Conditions (A) RKO cells were treated as indicated. Hu (6 hr) was used in either normoxia (21% O_2_) or hypoxia (2% O_2_). WB was carried out; β-tubulin was the loading control. (B) RKO cells were exposed to the O_2_ concentrations indicated for a period of 6 hr. Hu was added where indicated for 6 hr. Cells exposed to IR (5 Gy) were lysed immediately after IR. Shown is the % comet tail DNA. Data are represented as mean ± SEM. (C) p53^−/−^ MEFs with overexpressed mutant K-Ras or GFP were exposed to Norm (21% O_2_) or Hyp (2% O_2_, 6 hr). WB was carried out as indicated. (D) RS in the absence of DNA damage induced by either hypoxia (<0.1% O_2_), oncogene expression, or Hu treatment leads to ATM activation and downstream signaling only in the hypoxia-induced chromatin context. As demonstrated, one of the critical components of this chromatin context is H3K9me3. Once active, ATM is involved in maintaining DNA replication. See also Figure S4.

Our data demonstrate that ATM responds to RS when it occurs in hypoxic cells enriched for H3K9me3. This model suggests that exposure to alternative sources of RS in an environment including increased H3K9me3 would also induce ATM activity. To address this, we used a combination of 2% O_2_ (because it would lead to H3K9me3 in the absence of RS) and Hu. Importantly, acute Hu treatment in normoxia does not increase H3K9me3 levels either globally (Figure 4A) or at the replication fork (Figure S4A). As predicted, neither 2% O_2_ alone nor a 6 hr exposure to Hu in normoxia induced ATM-S1981 or KAP-1-S824 phosphorylation. In contrast, a notable induction of both ATM-S1981 and KAP-1-S824 was seen when the Hu-treated cells were incubated at 2% O_2_ (Figure 4A). As expected, the combination of Hu and 2% O_2_ had led to RS, as shown by increased numbers of RPA32 foci (Figures S4B and S4C), but without resulting in the accumulation of DNA damage (Figure 4B). In order to further investigate the finding that RS can induce ATM activity in a hypoxic chromatin context, we made use of an alternative method to induce RS. We asked whether oncogene-induced RS in hypoxic conditions, in the absence of DNA damage, might lead to an enhanced DDR activation. Mutant K-Ras was overexpressed in cells exposed to normoxia or hypoxia (2% O_2_) for 6 hr (Figures S4D and S4E). Consistent with our hypothesis, we only observed significant ATM phosphorylation when mutant K-Ras was overexpressed in p53^−/−^ primary MEFs in hypoxic (2% O_2_) conditions (Figure 4C). The p53^−/−^ background was chosen to avoid the entry of cells into senescence upon overexpression of the oncogene. Collectively, these data support a model whereby ATM only responds to RS in the absence of DNA damage if it occurs in a chromatin context associated with hypoxia and specifically increased H3K9me3 (Figure 4D). These data suggest that hypoxia and RS may work together in mediating an early barrier to tumorigenesis, and that such a barrier may be initiated by ATM activation even in the absence of detectable damage.

## Discussion

Hypoxia (<0.1% O_2_) induces ATM activity in response to RS in a context that includes elevated H3K9me3. The induction of ATM in these specific situations pointed to a potential role for ATM in resolving RS. In response to the absence or inhibition of ATM activity, hypoxic S phase cells accumulated significant levels of DNA damage. This could suggest a kinase-dependent role for ATM in protecting replication structures in areas rich in factors associated with heterochromatin. A possible mechanism for this could be through hypoxia-induced ATM-dependent phosphorylation of KAP-1, which has a characterized role in chromatin relaxation (Goodarzi et al., 2011; Ziv et al., 2006). The pan-nuclear localization of ATM and KAP-1 phosphorylation in hypoxia could reflect the need to globally facilitate chromatin relaxation given the widespread localization of H3K9me3 in hypoxia (Figure 2C). Alternatively, ATM-mediated signaling to additional targets may also be relevant, such as CHK1/CHK2. We have shown previously that in hypoxic conditions, CHK1 phosphorylates and inactivates TLK1, which has a role in repressing the histone chaperone ASF1 (Groth et al., 2003; Pires et al., 2010). Interestingly, in conditions of RS, ASF1 buffers evicted H3K9me3 and, therefore, loss of ASF1 during hypoxia-induced RS could contribute to the increased levels of H3K9me3 that we observed around replication forks (Jasencakova et al., 2010). Most interestingly, we determined that inhibition of ATM activity in hypoxic conditions led to decreased DNA replication rates. Together, these data demonstrate a stress-specific role for ATM in facilitating DNA replication during RS.

In addition, we have identified a stress-responsive role for ATM in replication restart. Inhibition of ATM increased origin firing during reoxygenation-induced replication restart whereas having no effect on normal replication. Given that no new origins are licensed once cells have entered S phase, the increase in origin firing following ATM inhibition likely reflects an increase in the firing of dormant origins (Ge and Blow, 2010). This would suggest a role for ATM in regulating dormant origin firing in response to replication restart following RS in the absence of DNA damage.

We observed a global increase in H3K9me3 occurring in response to both 2% as well as <0.1% O_2_. This increase, however, was more prominent in response to <0.1% O_2_, and coincided with increased protein levels of some of the principal H3K9 methyltransferases. MDM2 has been shown to posttranslationally downregulate methyltransferases such as Suv39h1 (Mungamuri et al., 2012). It is possible that decreased MDM2 expression in hypoxia contributes to the stabilization of some of the histone methyltransferases under these conditions (Alarcón et al., 1999). Furthermore, the decreased activity of the 2-oxoglutarate-dependent family of histone demethylases under hypoxic conditions undoubtedly contributes to the hypoxia-induced H3K9me3, although this was not investigated in this study (Sánchez-Fernández et al., 2013). We also observed that hypoxia-induced H3K9me3 is enriched on PP2A-C. These data indicate that H3K9me3 could serve to negatively regulate PP2A following hypoxia-induced RS, thereby contributing to ATM phosphorylation and activation. It is also possible that ATR-mediated signaling contributes to hypoxia-induced ATM activation (Stiff et al., 2006).

Recent reports have demonstrated a role for TIP60 in facilitating ATM activation particularly in regions of heterochromatin in response to IR (Kaidi and Jackson, 2013; Sun et al., 2009). However, Price and colleagues showed a dependency on MRN to recruit the ATM-TIP60 complex to sites of irradiation-induced DSBs. Given the absence of DSBs and the lack of requirement for the MRN complex in ATM activation in hypoxia, it seems unlikely this mechanism is involved in hypoxia-induced ATM activation (Bencokova et al., 2009; Cam et al., 2010).

Our model supports the theory that the chromatin context in which RS occurs can strongly influence the DDR. A role for Suv39h1 in preventing Ras-induced lymphoma development in vivo has already been demonstrated, supporting the concept that Suv39h1/2-mediated regulation of ATM activity in a hypoxic context could halt tumorigenesis in response to oncogenic stress (Braig et al., 2005). We propose that the hypoxia-induced chromatin context could have a role to play in facilitating the activation of the DDR (and in particular ATM) in response to RS before DNA damage accumulates. Therefore, cells proliferating at an increased rate due to oncogenic activation in a hypoxic environment would activate ATM independent of the levels of DNA damage. This implies that the barrier to tumorigenesis induced by the DDR in hypoxic preneoplastic lesions could occur earlier than predicted and, most importantly, before the accumulation of damage in response to oncogene activation (Bartkova et al., 2005; Gorgoulis et al., 2005). The hypoxia-dependent induction of the DDR early in tumorigenesis will significantly contribute to the selective pressure to lose/mutate genes such as ATM or p53 and therefore drive development of therapy-resistant disease (Graeber et al., 1996; Squatrito et al., 2010).

## Experimental Procedures

Cell lines, treatments, and details of siRNA and oligonucleotide sequences used are available in the Supplemental Experimental Procedures. Immunoblotting and immunofluorescence were carried out as previously described (Bencokova et al., 2009). Comet assays, DNA fiber analysis, and iPOND analysis were carried out as described previously in Parsons et al. (2012), Pires et al. (2010), and Sirbu et al. (2012), respectively, with modifications to allow hypoxic/reoxygenation treatments. Detailed experimental procedures are available in the Supplemental Experimental Procedures.

## Supplemental Information

Document S1. Figures S1–S4 and Supplemental Experimental Procedures
