# Supplementary material for: Replication Stress and Chromatin Context Link ATM Activation to a Role in DNA Replication
Source: Mol Cell. 2013 Dec 12;52(5):758–66. doi: 10.1016/j.molcel.2013.10.019 (PMC3898930; doi:10.1016/j.molcel.2013.10.019)
Supplement: Document S1. Figures S1–S4 and Supplemental Experimental Procedures [file mmc1.pdf]

Molecular Cell, Volume *52*

Supplemental Information

Replication Stress and Chromatin Context
Link ATM Activation to a Role in DNA Replication

Monica M. Olcina, Iosifina P. Foskolou, Selvakumar Anbalagan, Joana M. Senra, Isabel M. Pires, Yanyan Jiang, Anderson J. Ryan, and Ester M. Hammond

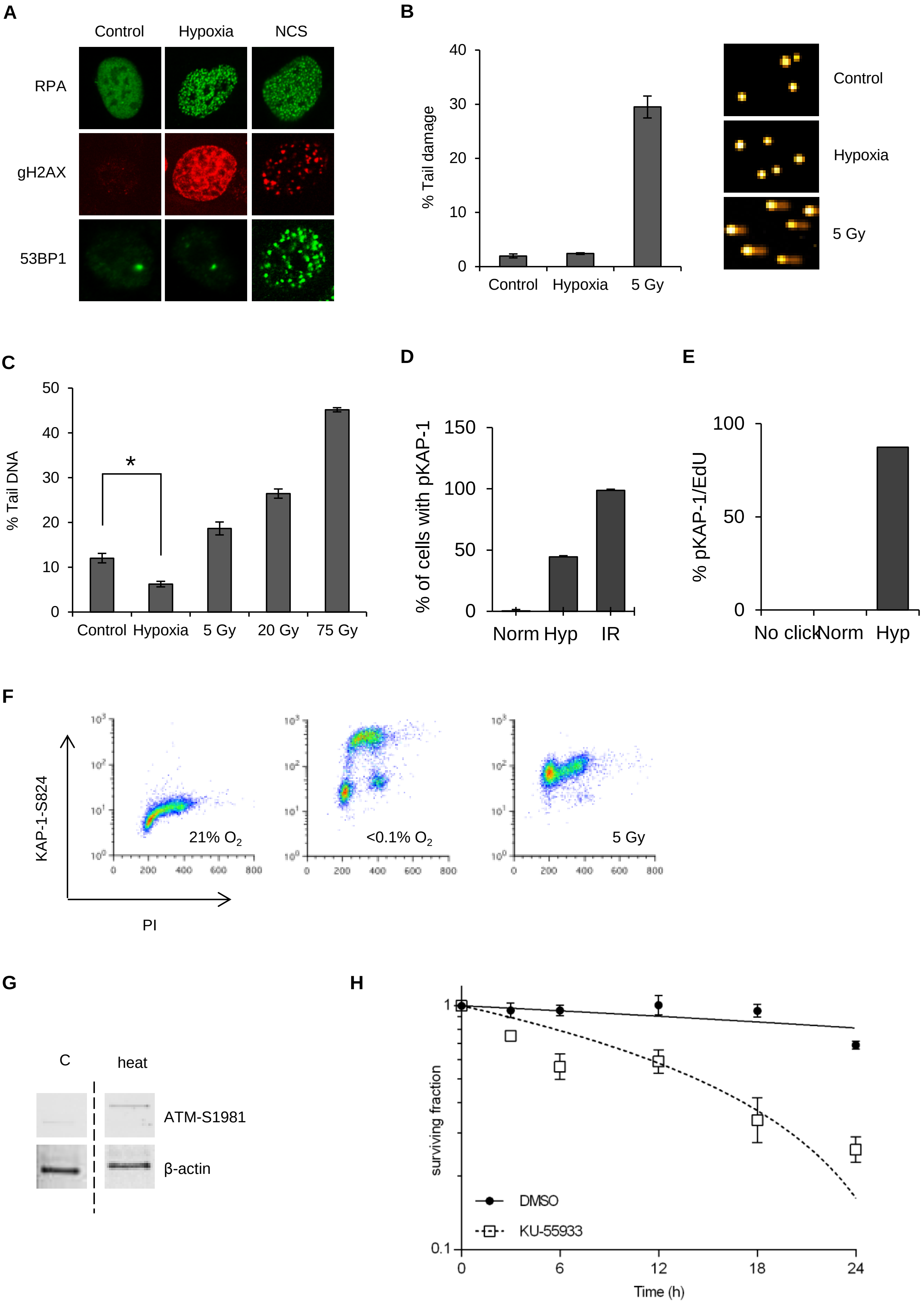
A
Control
Hypoxia
NCS
RPA
gH2AX
53BP1
B
% Tail damage
Control
Hypoxia
5 Gy
C
% Tail DNA
*
Control
Hypoxia
5 Gy
20 Gy
75 Gy
D
% of cells with pKAP-1
Norm
Hyp
IR
E
% pKAP-1/EdU
No click
Norm
Hyp
F
KAP-1-S824
PI
21% O2
<0.1% O2
5 Gy
G
C
heat
ATM-S1981
β-actin
H
surviving fraction
Time (h)
DMSO
KU-55933

Figure S1, (relating to Figure 1). **Exposure to hypoxia (<0.1% O_2) leads to ATM-mediated signaling in S-phase in the absence of detectable DNA damage. Inhibition of ATM activity leads to increased loss of viability in hypoxia**

(A) RKO cells were exposed to hypoxia (<0.1% O_2) for 6 h or NCS (200 ng/ml – 6 h). Cells were then fixed and stained as indicated. The hypoxic cells were fixed in hypoxic conditions with equilibrated solutions to avoid reoxygenation. **(B)** Quantification of the % comet tail DNA after alkaline comet assay for each condition is shown next to representative images. **(C)** RKO cells treated as indicated and processed for neutral comet assay (hypoxia <0.1% O_2 for 6 h). Quantification of the % comet tail DNA for each condition is shown. **(D)** RKO cells were treated with either Norm (21% O_2); Hyp (6 h, <0.1% O_2) or IR (1 Gy, cells fixed 30 minutes post IR); stained and scored for KAP-1 phosphorylation. pKAP-1= KAP-1-S824. At least 100 cells were measured for each of three independent experiments. **(E)** RKO cells were labeled with EdU for the times indicated and co-stained with KAP-1-S824. Norm (21% O_2), Hyp (<0.1% O_2 - 6 h). Quantification of the pKAP-1/EdU co-staining is shown. **(F)** RKO cells were treated with either Norm (21% O_2); Hyp (6 h, <0.1% O_2) or IR (5 Gy, cells fixed immediately post IR). Cells were then stained for KAP-1-S824 and for DNA content using propidium iodide (PI) and analysed using FACS. **(G)** Treatment with heat leads to the induction of ATM phosphorylation. RKO cells were exposed to 42ºC for either 0 or 1 h and Western blotting (WB) was carried out with the indicated antibodies. **(H)** Inhibition of ATM activity leads to increased loss of viability in hypoxia. RKO cells were treated with DMSO or KU-55933 and exposed to Norm (21% O_2) or Hyp (<0.1% O_2) for the indicated time periods. Clonogenic survival assays were carried out. Data are represented as mean +/- SEM.

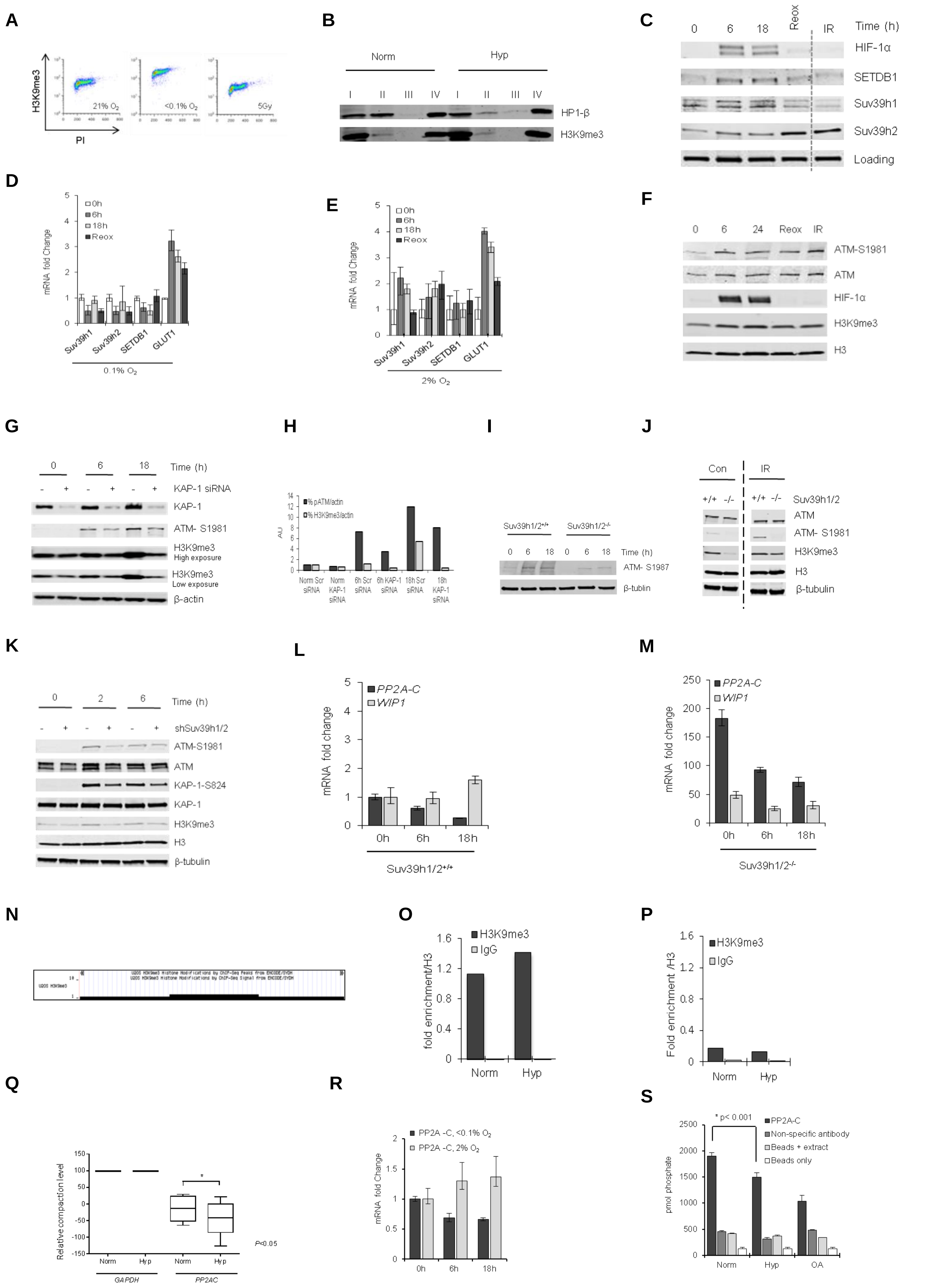

A
H3K9me3
PI
21% O2
<0.1% O2
5Gy
B
Norm
Hyp
I II III IV I II III IV
HP1-β
H3K9me3
C
0 6 18 Reox IR Time (h)
HIF-1α
SETDB1
Suv39h1
Suv39h2
Loading
D
0h
6h
18h
Reox
mRNA fold Change
Suv39h1 Suv39h2 SETDB1 GLUT1
0.1% O2
E
0h
6h
18h
Reox
mRNA fold Change
Suv39h1 Suv39h2 SETDB1 GLUT1
2% O2
F
0 6 24 Reox IR
ATM-S1981
ATM
HIF-1α
H3K9me3
H3
G
0 6 18 Time (h)
KAP-1 siRNA
KAP-1
ATM- S1981
H3K9me3 High exposure
H3K9me3 Low exposure
β-actin
H
% pATM/actin
% H3K9me3/actin
AU
Norm Scr siRNA
Norm KAP-1 siRNA
6h Scr siRNA
6h KAP-1 siRNA
18h Scr siRNA
18h KAP-1 siRNA
I
Suv39h1/2+/+
Suv39h1/2-/-
0 6 18 0 6 18 Time (h)
ATM- S1987
β-tublin
J
Con
IR
+/+ -/- +/+ -/- Suv39h1/2
ATM
ATM- S1981
H3K9me3
H3
β-tubulin
K
0 2 6 Time (h)
shSuv39h1/2
ATM-S1981
ATM
KAP-1-S824
KAP-1
H3K9me3
H3
β-tubulin
L
PP2A-C
WIP1
mRNA fold change
0h 6h 18h
Suv39h1/2+/+
M
PP2A-C
WIP1
mRNA fold change
0h 6h 18h
Suv39h1/2-/-
N
U2OS H3K9me3
O
H3K9me3
IgG
fold enrichment/H3
Norm Hyp
P
H3K9me3
IgG
Fold enrichment /H3
Norm Hyp
Q
Relative compaction level
Norm Hyp Norm Hyp
GAPDH
PP2AC
P<0.05
R
PP2A -C, <0.1% O2
PP2A -C, 2% O2
mRNA fold Change
0h 6h 18h
S
* p< 0.001
PP2A-C
Non-specific antibody
Beads + extract
Beads only
pmol phosphate
Norm Hyp OA

Figure S2 (relating to Figure 2). **Hypoxia-induced chromatin changes. Hypoxia-induced H3K9me3 is required for ATM activity**

(A) RKO cells were treated with either Norm (21% O_2); Hyp (6 h, <0.1% O_2) or IR (5 Gy, cells fixed immediately post IR). Cells were then stained for H3K9me3 and for DNA content using propidium iodide (PI) and analysed using FACS. **(B)** Chromatin fractionation experiments were carried out with RKO cells exposed to Norm (21% O_2) or Hyp (<0.1% O_2 - 6 h). Fraction I corresponds to the whole cell extract (WCE) and IV corresponds to the most stringent nuclear fraction. Shown is a WB with H3K9me3 used as a nuclear fraction control. **(C)** RKO cells were exposed to 0, 6, 18 h of <0.1% O_2, Reox (6 h, <0.1% O_2 followed by 1 h of 21% O_2); or IR (5 Gy, cells harvested 30 minutes post-IR). WB was carried out. Quantification of bands (as fold induction): SETDB1: 6 h = 6.46, 18 h = 4.57, Reox = 0.87; Suv39h2: 6 h =1.39, 18 h = 1.75, Reox = 1.96, IR = 1.81; Suv39h1: 6 h =0.78, 18h = 1.16, Reox = 0.81, IR = 0.36. **(D)** The mRNA levels of Suv39h1, Suv39h2, SETDB1 and as control GLUT1 were measured by qRT-PCR in RKO cells exposed to either <0.1% O_2 or **(E)** 2% O_2. The reox sample was exposed to the relevant oxygen concentration for 6 h and then 21% O_2 for 2 h. **(F)** Calu6 cells were exposed to 0, 6, 24 h of <0.1% O_2, Reox (6 h, <0.1% O_2 followed by 1 h of 21% O_2); IR (5 Gy, cells fixed 30 minutes post IR). WB was carried out. **(G)** RKO cells were treated with KAP-1 or Scramble siRNA and exposed to 0, 6, 18 h of <0.1% O_2. WB was carried out as indicated. The low and high exposure of H3K9me3 bands is also shown. **(H)** Quantification of the pATM/actin and H3K9me3/actin ratio. pATM = ATM-1981. **(I)** Suv39h1/2$^{+/+}$ and Suv39h1/2$^{-/-}$ MEFs were treated as indicated. The ATM-S1987 antibody used was produced from a synthetic peptide corresponding to amino acids 1974-1988. β-tubulin = loading control. **(J)** Suv39h1/2$^{+/+}$ and Suv39h1/2$^{-/-}$ MEFs were treated as indicated. WB was carried out. IR = 5 Gy, cells harvested immediately post IR. β-tubulin = loading control. **(K)** RKO cells were treated with or without shSuv39h1/2 and exposed to 0, 2 or 6 h of <0.1% $O_{2.}$ WB was carried out. **(L)** The mRNA levels of WIP1 and PP2A-C were assessed by qRT-PCR in Suv39h1/2$^{+/+}$ and (**M**) Suv39h1/2$^{-/-}$ MEFs following exposure to hypoxia (<0.1% O_2) for the times indicated. Fold change is expressed relative to the level of expression in Suv39h1/2$^{+/+}$ cells. **(N)** UCSC genome browser profile of the region of PP2A-C with moderate H3K9me3 binding. **(O)** RKO cells were exposed to either Norm (21% O_2) or Hyp (<0.1% O_2 - 6 h). The enrichment of H3K9me3 at the loci on PP2A-C was assessed by ChIP. A representative graph from three independent experiments is shown. **(P)** RKO cells were exposed to either Norm (21% O_2) or Hyp (<0.1% O_2 - 6h). The enrichment of H3K9me3 on PP2A-C was assessed by ChIP. Primers were designed to target a region of minimal H3K9me3 enrichment. **(Q)** RKO cells were exposed to 0 or 6 h of <0.1% O_2. Analysis of chromatin compaction levels was performed using the EpiQ assay. Chromatin accessibility was compared to the constitutively accessible *GAPDH* region. *=p<0.05. **(R)** mRNA levels of *PP2A-C* were assessed following exposure of RKO cells to either 0, 6, 18 h of hypoxia (<0.1 or 2% O_2). mRNA levels for all qRT-PCR were normalized to *18S* and expressed as fold change. Error bars indicate error between technical replicates. **(S)** Phosphatase activity of PP2A-C was determined following treatment of RKO cells with either Norm (21% O_2), Hyp (<0.1% O_2 - 6 h) or Okadaic acid (OA) (2 h). GAPDH (non-specific antibody), beads plus extract and beads only were used as controls. A representative graph from one of three independent experiments is shown. Error bars indicate the error between technical replicates for the represented experiment. Data are represented as mean +/- SEM.

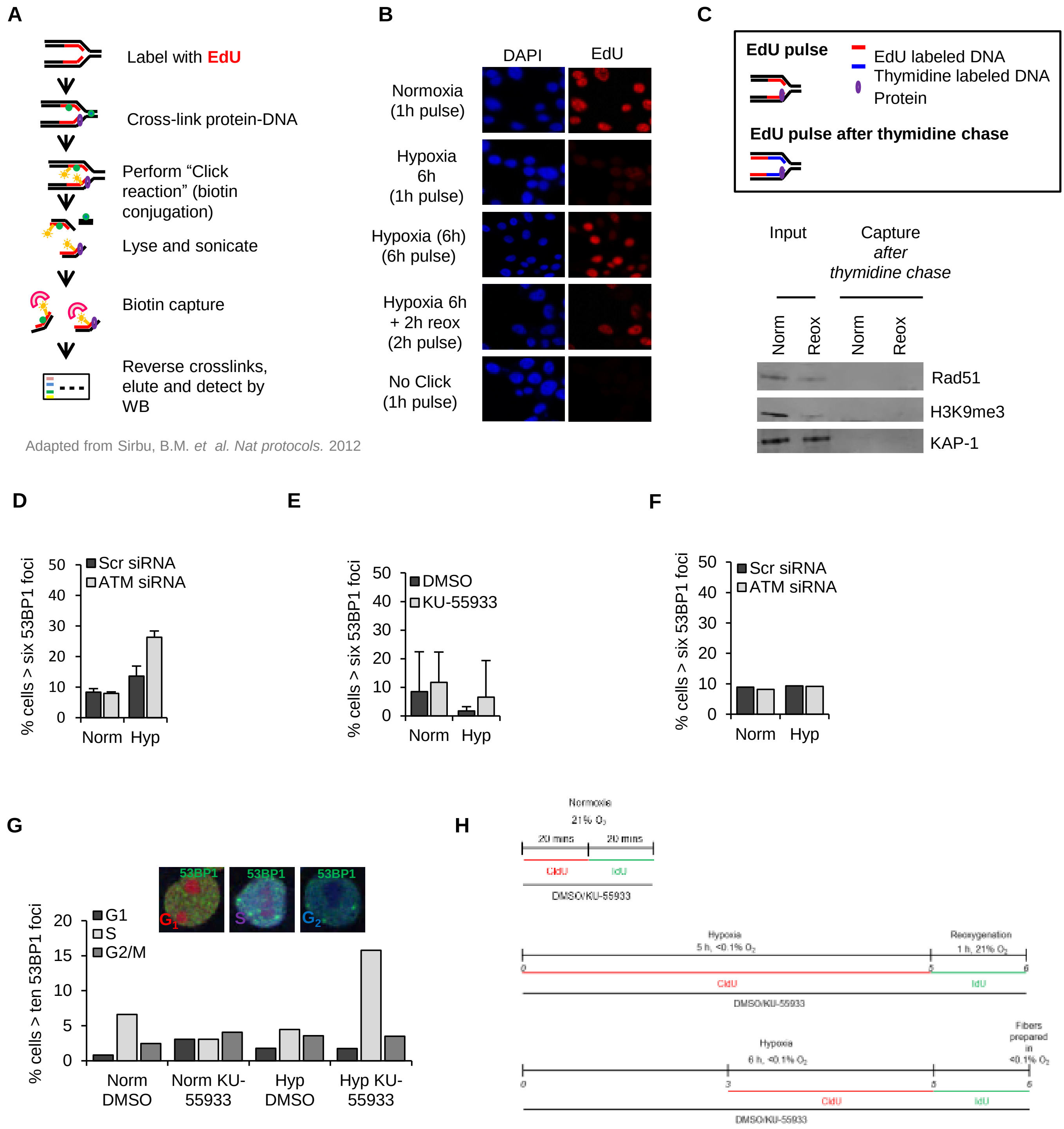

I

	Norm (IdU kb/min)	Hyp (IdU kb/min)
Scr siRNA	0.22 ± 0.08	0.13 ± 0.03
ATM siRNA	0.20 ± 0.4	0.05 ± 0.01
	ns	p<0.05

J

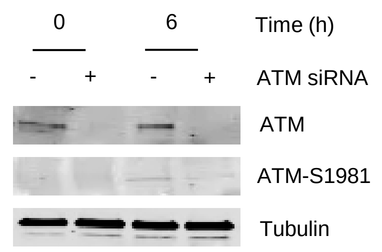

Figure S3 (relating to Figure 3). **Hypoxia induced ATM has a role in replication** **(A)** A schematic representation of the iPOND experiment performed with the three experimental conditions tested is shown. **(B)** EdU labeling times were optimized to ensure approximately equal amounts of EdU were incorporated in each condition. Shown are representative images for the EdU labeling times chosen. For hypoxic treatments 1 hour of EdU incorporation results in minimal EdU incorporation due to the hypoxia-induced replication stress. 6 hours of EdU incorporation in hypoxia results in a similar level of incorporation as seen following 1 hour of EdU labeling under normoxic conditions. Following reoxygenation from hypoxia-induced replication stress 2 hours of EdU labeling results in a comparable level of EdU incorporation to 1 hour labeling in normoxic conditions. (**C**) Schematic representation of the thymidine chase experiment. iPOND was carried out following exposure of cells to either Norm (21% O_2) or Reox (6 h, <0.1% O_2 followed by 2 h of 21% O_2). Thymidine chase was performed for the last 30 minutes of each condition. **(D)** RKO cells were treated with Scramble (Scr) or ATM siRNA and exposed to Norm (21% O_2) or Hyp (<0.1% O_2 - 6 h). The quantification of 53BP1 foci counted in two independent experiments is shown. P value between Norm Scr and Hyp Scr = >0.05. **(E)** RKO cells were treated with DMSO or KU-55933 and exposed to Norm (21% O_2) or Hyp (2% O_2 - 6 h). The quantification of 53BP1 foci counted in two independent experiments is shown. **(F)** RKO cells were treated as in (E) but in the presence of Scr or ATM siRNA. The quantification of 53BP1 foci counted in one experiment is shown. **(G)** H1299 cells (labeled with Fucci plasmids) were exposed to Norm (21% O_2) or Hyp (<0.1% O_2 - 6 h) in the presence of DMSO or KU-55933. The quantification of 53BP1 foci counted in each cell cycle phase is shown together with a representative image of cells in each phase. **(H)** Schematic representation of DNA fiber analysis in normoxia, after reoxygenation and in hypoxia (<0.1% O_2). **(I)** The replication rates of cells treated with Scramble or ATM siRNA and exposed to Norm (21% O_2) or Hyp (<0.1 % O_2 - 6 h) were scored. An average of the average IdU-incorporation rates for each of three independent experiments are shown for each condition. ns = non significance. **(J)** Spare cells collected following DNA fiber spreading were lysed and WB with the indicated antibodies was carried out to validate the level of ATM knockdown. Data are represented as mean +/- SEM.

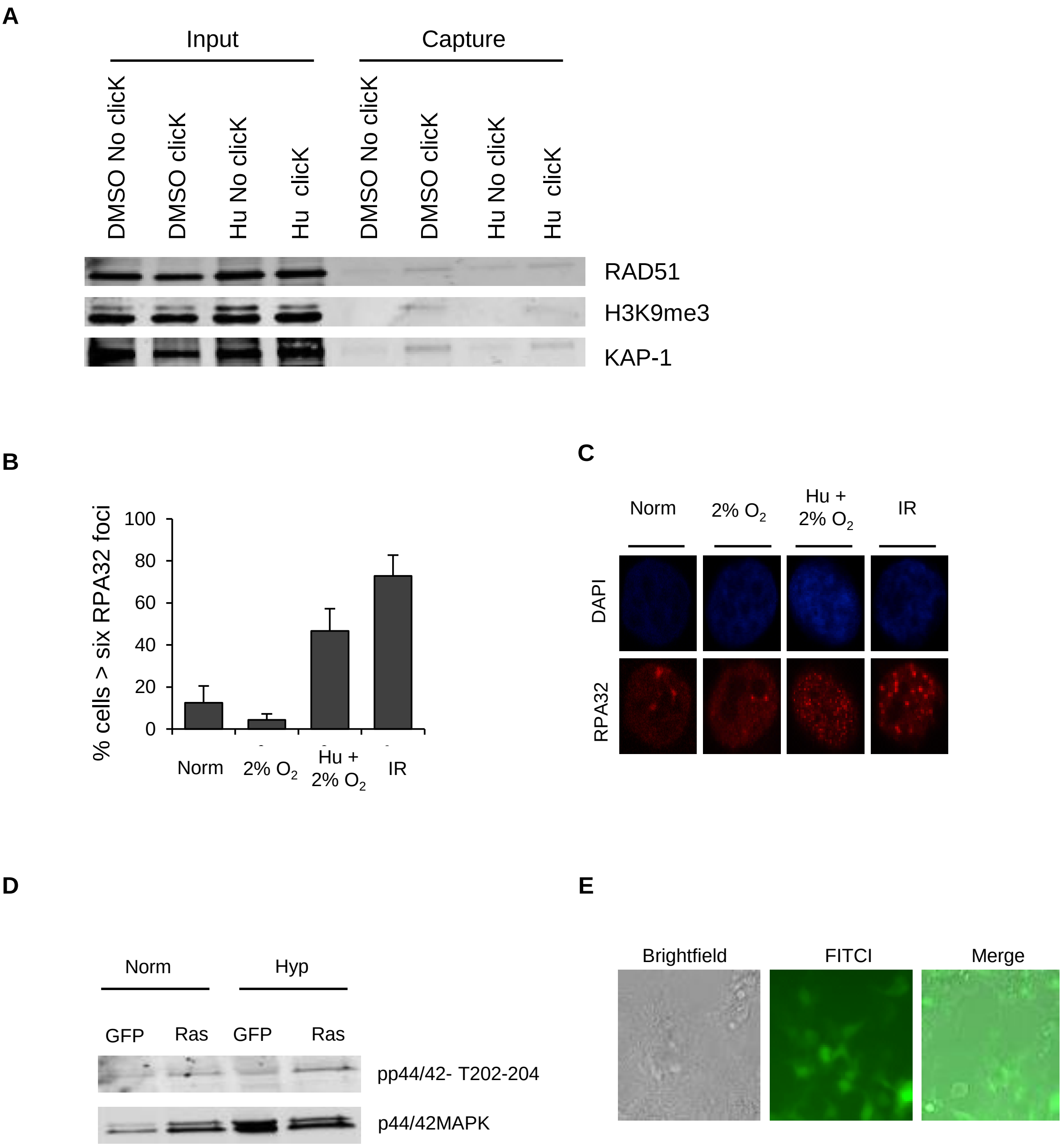

Figure S4 (relating to Figure 4). **Both Hu and oncogene-induced replication stress leads to ATM activation in hypoxic conditions**
(A) H3K9me3 is not enriched around the replication fork following Hu-induced replication stress**.** iPOND was carried out following exposure of cells to either DMSO or Hu (6 h). EdU labeling times = 30 minutes for all conditions. **(B)** RKO cells were treated as indicated. Norm (21% O_2); 2% O_2 - 6 h; Hu + 2% O_2 (6 h of Hu in 2% O_2) and IR (5 Gy, cells harvested 30 minutes post IR). Quantification of RPA32 foci counted from three independent experiments is shown. At least 100 cells were counted per condition. Data are represented as mean +/- SEM **(C)** Representative image of cells counted.

Figure S4 (relating to Figure 4) continued. **(D)** Mutant K-Ras or GFP was overexpressed in p53$^{-/-}$ MEFs. These cells were then exposed to Norm (21% O_2) or Hyp (2% O_2 – 6 h). The levels p44/p42-T202-Y204 MAPK and total p44/p42 MAPK were assessed by WB. **(E)** As a transfection control, expression of GFP was assessed by microscopy. A representative image is shown.

EXPERIMENTAL PROCEDURES

Cell lines and treatments. RKO (human colorectal), D15 Suv39h1/2$^{-/-}$, W9 Suv39h1/2$^{+/+}$ MEFs (Thomas Jenuwein, Freiburg, Germany), HEK 293T, NIH 3T3, p53$^{-/-}$MEFs (Xin Lu, Oxford, UK) and H1299 (expressing Fucci plasmids), cells were grown in DMEM with 10% FBS, in a standard humidified incubator at 37°C and 5% CO_2 (Peters et al., 2001). The media in which the pair of D15 Suv39h1/2$^{-/-}$ and W9 Suv39h1/2$^{+/+}$ MEFs were grown was also supplemented with 0.1 mM β-mercaptoethanol. All cell lines were routinely mycoplasma tested and found to be negative. Doxorubicin (Dox) was used at a concentration of 2 μM for 4 hours. Hydroxyurea (Hu) was used at a concentration of 1 mM. ATM inhibitor, KU-55933, (2-morpholin-4-yl-6-thianthren-1-yl-pyran-4-one) (Calbiochem) was used at 10 μM. Okadaic acid (OA) (Sigma-Aldrich) was used at a concentration of 0.5 μM for 2 hours. EdU (5-ethynyl-2'-deoxyuridine) and thymidine were used at a concentration of 10 μM.

Hypoxia treatment. Hypoxia treatments were carried out in a Bactron II anaerobic chamber (Shell labs) or an In vivo$_2$ 400 (Ruskinn) (for oxygen concentrations between 2-0.2%). For experiments at <0.1% O_2 cells were plated on glass dishes. Unless the experiment involved periods of reoxygenation cells were harvested inside the chamber with equilibrated solutions. Oxygen concentrations were periodically verified using an Oxylite probe (Oxford Optronix, UK).

Irradiation. All irradiations were carried out using a Gamma Service® GSR D1 irradiator containing a Cs137 source. The dose rates of the system, as

determined by the supplier, were 1.938 Gy/min and 1.233 Gy/min depending on the distance from the source.

Immunoblotting*.* Cells were lysed in UTB (9 M urea, 75 mM Tris-HCl pH 7.5 and 0.15 M β-mercaptoethanol) and sonicated briefly. Antibodies used were CHK2-T68, p53-S15, H3K9me2, KAP1-S824, (Bethyl), ATM-S1981 (Epitomics), ATM-S1981(Rockland), β-actin (Santa Cruz), γH2AX and H3K9me3 (Upstate), HIF-1α BD Biosciences), β-tubulin, H3, H3K27me3 and HP1β (Abcam), pp44/42 T202-Y204 MAPK and p44/p42 MAPK (Cell Signaling). The Odyssey infrared imaging technology was used (LI-COR Biosciences). In each case experiments were carried out in triplicate and a representative blot is shown unless otherwise stated.

Immunofluorescence*.* Staining was carried out as previously described (Bencokova et al., 2009). 53BP1 (Novus Biologicals), RPA32 (Cell Signaling), KAP1-S824, (Bethyl), ATM-S1981 (Epitomics) and γH2AX and H3K9me3 (Upstate) were used. Due to the presence of 53BP1 foci in the nuclei of unstressed cells induction of DNA damage was quantified by counting cells with more than 6 foci. At least 100 cells were counted per condition unless otherwise stated. Mitotic cells were collected by mitotic shake-off, replated and treated while in G_1 phase (Hammond et al., 2002). EdU labeling and co-staining was carried out according to the manufacturer's instructions (Invitrogen). Cells were visualized using a Bio-Rad Radiance or LSM780 (Carl Zeiss Microscopy Ltd) confocal microscope.

Alkaline Comet Assay. Comet assays were performed as previously (Parsons et al., 2012), with modifications to allow hypoxic treatments. All

solutions used for hypoxic treatment were equilibrated at indicated oxygen tensions. 6.5 x 10^4 cells were seeded and allowed to adhere for 12 hours, before exposing them to the indicated treatments. Treated cells were trypsinized and embedded in 1% low-melting agarose. Lysis buffer (2.5 M NaCl, 100 mM EDTA disodium salt, 10 mM Tris base, pH 10.5) was then added for an hour at room temperature in the dark. For hypoxic samples lysis was carried out under hypoxic conditions. Slides were removed and washed, followed by incubation in cold electrophoresis buffer (300 mM NaOH, 1 mM EDTA and 1% DMSO, pH > 13) for 30 minutes. Electrophoresis was carried out at 25 V, 300 mA for 25 minutes. Slides were stained with SYBR gold (Invitrogen) after neutralization with 0.5 M Tris-HCl (pH 8.0). Comets were analyzed using Komet 5.5 image analysis software (Andor Technology). 50 cells were counted per slide. Two slides were counted per condition for each experiment.

siRNA transfection. ATM siRNA (sequence: GCGCAGUGUAGCUACUUCUUCUAUU) (Invitrogen) or Stealth RNAi negative control (Invitrogen) were transfected into RKO cells using Dharmafect (Thermo Scientific) at a final concentration of 50 nM; according to the manufacturers' instructions. Cells were harvested 72 hours post-transfection.

Retroviral transductions. MEFs were transduced with pBABE-Puro GFP, or pBABE-Puro K-ras-V12 (Addgene plasmid 9052) (gift from Eric O'Neill, Oxford, UK) as previously described (Palmero and Serrano, 2001). In brief, HEK 293T packaging cells were transfected with pCL-Eco helper vector and the expressing retroviral vector of choice. 24 hours post-transfection the cell

culture medium was replaced with fresh medium. The following day recipient early passage non-transformed p53$^{-/-}$ MEFs were infected with retroviral supernatants. Two additional infections were carried out 24 and 32 hours later. 24 hours after the last infection, cells were washed and grown in medium containing 2 μg/ml puromycin.

Identification of Proteins on Nascent DNA (iPOND). This procedure was carried out as previously described with some modifications (Sirbu et al., 2012). In brief, 6-7 x 10^8 cells were used per condition. For cells exposed to hypoxia EdU (10 μM) was added immediately before placing the cells in the chamber. Those cells allowed to reoxygenate before crosslinking and harvesting were treated with EdU as soon as they were taken out of the chamber and for the whole duration of the reoxygenation period (2 hours). For cells exposed to normoxia EdU was added for 1 hour before crosslinking and harvesting (Figure S3).

Single-molecule DNA fiber analysis*.* DNA fibers were generated as previously described (Pires et al., 2010; Wilsker et al., 2008). Images of fiber spreads were taken by using a Bio-Rad Radiance or LSM780 (Carl Zeiss Microscopy Ltd) confocal microscope and analyzed using ImageJ software (NIH). At least 50-100 tracks were scored per condition.

Xenograft study. Calu-6 (ATCC HTB-56) human non-small cell lung cancer cells (0.1 mL, 1 x 10^6) were injected subcutaneously into female BALB/C nude mice (6-8 weeks old) at a single dorsal site. Growing tumors (100-400 mm^3) were formalin fixed and paraffin embedded.

Immunohistochemistry staining (IHC). IHC analysis was performed on 4 µm sections of paraffin-embedded Calu6 xenografts as described with a few modifications (Bottini et al., 2000). Antigen retrieval was performed for 1 minute and 30 seconds at 125°C with citrate buffer in 0.05% PBS-tween (pH 8.0). Endogenous peroxidase activity was blocked using 3% H_2O_2 for 30 minutes at room temperature. Non-specific antibody binding was avoided by treating samples with protein blocking solution (Abcam) for 15 minutes before adding the diluted primary antibody overnight at 4°C. H3K9me3 (Abcam), CAIX (Adrian Harris, Oxford, UK) and ATM-S1981 primary antibodies were used. Slides were then incubated for 30 minutes with secondary EnVision System HRP-labeled polymer (Dako). Following this incubation slides were stained with diaminobenzidene (DAB) solution (DAB+, Dako) for 1 minute for H3K9me3 staining or 2 minutes for CAIX and ATM-S1981 staining. Samples were counterstained with hematoxylin for 30 seconds and mounted with DPX mounting media (Surgipath). Slides were imaged using the Aperio system at 20x magnification and quantified using Scanscope software. An intensity score of 2 or above represents high H3K9me3 or ATM-S1981 staining. Intensity scores of 1 or above represent CAIX positive regions.

Statistical analysis. Statistical significance of differences between means of n=3 experiments was determined using Student's *t*-test (p values indicated accordingly in figure legend or main text) unless otherwise stated. Error bars represent +/- SEM (Standard Error of the Mean) unless otherwise stated in each figure.

SUPPLEMENTAL EXPERIMENTAL PROCEDURES

Neutral comet assay. 0.65 x 10^5 RKO cells were plated in glass petri dishes and allowed to adhere for 12 hours. Cells were embedded as described for alkaline comet assay. Coated slides were submerged into lysis buffer pH 8.3 (30 mM EDTA, 0.5% sodium dodecyl sulphate, 0.5 mg/ml Proteinase-K) for 16 hours in the dark at 37°C (4°C for 2 h and then transferred to 37°C for IR studies). For hypoxic studies all the slides were incubated at 37°C inside the hypoxic chamber with pre-equilibrated solutions. Following lysis, the slides were rinsed with TBE buffer pH 8.5 (90 mM Tris, 90 mM Boric acid, 2 mM EDTA) for 16 hours and electrophoresed at 1.0 V/cm for 25 minutes, with fresh TBE buffer. Post electrophoresis, slides were washed with double distilled water and stained with SYBR gold (Invitrogen, S11494) for 30 minutes. Stained slides were washed with double distilled water and dried overnight and analysed for comet using the Komet 5.5 image analysis software (Andor Technology).

FACS analysis. For staining of KAP-1-S824 or H3K9me3 with propidium iodide (PI), RKO cells were placed in normoxia, hypoxia (<0.1% O_2 for 6 h) or irradiated with 5 Gy. Following treatment, cells were fixed with 4% paraformaldehyde, washed twice and permeabilized with 0.5% Triton-X for 20 min. Cells were then incubated with the primary antibody diluted 1:100 in 1% BSA in 1 x PBS for 60 min followed by the secondary antibody diluted 1:200 in 1% BSA in 1 x PBS for 60 min. Primary antibodies used were as follows: KAP1-S824 and H3K9me3 (Cell Signaling Technology). Secondary antibody used was Alexa 488-conjugated goat anti-rabbit (Molecular Probes). Finally

the DNA content was stained with propidium iodide and FACS analysis was carried out using a Becton Dickinson FACSort. Samples were later analysed using CellQuest Pro and ModFit LT software.

Chromatin immunoprecipitation (ChIP). ChIP was carried out as described (Bindra et al., 2005). 3 μg H3 and 6 μg H3K9me3 antibodies (Abcam) were used for immunoprecipitation. Rabbit pre-immune serum was used as an IgG control. A no antibody control sample was also included in each experiment. Following incubation of chromatin with Protein A-agarose for 1 hour, 100 μl from the no antibody sample were saved and used as input DNA. Collected beads were washed five times with the buffers indicated at the end of the supplemental information before elution from the beads. The collected input DNA was processed together with the other samples following elution. Reversal of cross-links was carried out by addition of 20 μL 5 M NaCl (per 500 μL of eluate) and incubation at 65°C overnight and digestion was performed by incubating samples with proteinase K in 1 M Tris pH 6.5 and 0.5 M EDTA at 45°C for 1 hour. Extracted DNA was dissolved in 25 μl water. qRT-PCR was carried out as described above with the primers stated in the supplemental information. Immunoprecipitation samples were diluted 1:6 and input was diluted 1:100 for qRT-PCR analysis. Fold enrichment is expressed as a % of input and is normalized to total H3 in each sample.

qRT-PCR. RNA was extracted using Trizol (Invitrogen/Life Technologies). Verso (Thermo Scientific) or SuperScript® VILO™ (Invitrogen/Life Technologies) cDNA synthesis kits were used to reverse transcribe cDNA from total RNA according to manufacturer's instructions. Quantitative real time PCR was performed as previously using the Thermo Scientific Verso™ qRT-

PCR following manufacturers recommendations (Pires et al., 2010). Reactions were carried out in a 7500 Fast real time PCR detection system (Applied Biosystems). mRNA fold change levels were normalized to *18S* ribosomal RNA and calculated using a $2^{-\Delta\Delta Ct}$ method. Error bars indicate the error between three technical replicates ± RQ_{max} and RQ_{min} from one experiment.

Phosphatase assay. This assay was carried out according to the manufacturer's instructions (Millipore) with a few modifications. In brief, extracts were prepared in such a way as to ensure minimal phosphate content of the extracts (Tanaka et al., 1992). Cell extracts were resuspended in one packed-cell volume of buffer I, containing 10 mM Tris-HCl (pH 7.8), 200 mM KCl, 1 mg/ml of protease inhibitor cocktail (Roche),1 mM PMSF and 1 mM NEM. Cells were then resuspended in two packed-cell volumes of Buffer II containing 10 mM Tris-HCl (pH 7.8), 600 mM KCl, 40% glycerol, 0.1 mM EDTA and 0.2% Nonidet P-40. The cell lysate was then centrifuged at 9800 x g at 4°C for 20 min and the supernatant was harvested and stored at 80°C for at least 30 minutes prior to immunoprecipitation with PP2A-C antibody (Upstate) and subsequent phosphatase activity measurement.

EpiQ Chromatin analysis

RKO cells were seeded at a concentration of 3 x 10^5 cells/ml in a 48-well plate, allowed to attach overnight and exposed to 6h of <0.1% O_2. Quantitative assessment of chromatin structure was performed using an EpiQ chromatin analysis assay (Bio-Rad Laboratories) according to the manufacturer's protocol. The qRT-PCR primers used as controls for

inaccessible (RHO) and accessible (GAPDH) chromatin regions were provided by the manufacturer and the PP2AC primers used were the same as for ChIP analysis.

Clonogenic survival assay. Cells were treated with either KU-55933 or DMSO (as a control). Colonies (of at least 50 cells) were allowed to form for 10-14 days. Colonies were then stained with crystal violet and counted.

Lentiviral transductions. shRNAs targeting Suv39h1 (pSMP-Suv39h1 5′GAGCTCACCTTTGATTACA-3′) (Addgene plasmid 36342), Suv39h2 (pSMP-Suv39h2 5′- CCCGTTACTGCTTCAGCAA-3′) (Addgene plasmid 36344) and a Luciferase control shRNA (pSMP-Luc 5′-CCCGCCTGAAGTCTCTGATTAA -3′) (Addgene plasmid 36394) were used (we thank George Daley for depositing these plasmids in Addgene). RKO cells were transduced with shRNA lentiviruses as previously described (Onder et al., 2012). Twenty-four hours after the last infection, cells were washed extensively and grown in medium containing 1 μg/ml puromycin.

Biochemical fractionation. Fractionation was carried out as described in (Andegeko et al., 2001). Fractionation was carried out in four consecutive steps with the supernatant being collected at each stage. Fractions I and II were fractionated in Nonidet P-40 buffer (containing 50 mM HEPES, pH 7.5, 150 mM NaCl, 1 mM EDTA, 0.2% Nonidet P-40). 0.5% Nonidet P-40 buffer was used to fractionate fraction III. Protease (complete EDTA free; Roche) and phosphatase (Phospho-Stop; Roche) inhibitors were added to all buffers. For fraction IV, harvested cells were lysed in 1 X SDS-PAGE sample buffer. Equal aliquots of each fraction corresponding to 1/5 th of the total sample

volume were separated on 7.5 or 12% sodium dodecyl sulphate gels, and western blotting was carried out.

ChIP Buffers:

SDS cell lysis buffer

Final	Stock	Per ml	Per 50 ml
1% SDS	20%	50 μl	2.5 ml
10 mM EDTA	0.5 M	20 μl	1ml
50 mM Tris pH8.1	1 M	50 μl	2.5 ml
25x Complete Pi	25X	40 μl	2 ml
Milli Q H_2O	-	840 μl	43 ml

ChIP dilution buffer

Final	Stock	Per 50 ml
0.01% SDS	20%	25 μl
1.1% Triton X-100	100%	550 μl
1.2 mM EDTA	0.5 M	120 μl
16.7 mM Tris pH8.1	1 M	835 μl
167 mM NaCl	5 M	1.67ml
25x Complete Pi	25X	2 ml
Milli Q H_2O	-	Up to 50 ml

Low salt wash solution

Final	Stock	Per ml	Per 50ml
0.1% SDS	20%	5 μl	250 μl
1% Triton X-100	100%	10 μl	500 μl
2 mM EDTA	0.5 M	4 μl	200 μl
20 mM Tris pH8.1	1 M	20 μl	1 ml
150 mM NaCl	5 M	30 μl	1.5 ml
Milli Q H_2O	--	931μl	Up 50 ml

High salt wash solution

Final	Stock	Per ml	Per 50 ml
0.1% SDS	20%	5 μl	250 μl
1% Triton X-100	100%	10 μl	500 μl

2 mM EDTA	0.5 M	4 µl	200 µl
20 mM Tris pH8.1	1 M	20 µl	1 ml
500 mM NaCl	5 M	100 µl	5 ml
Milli Q H_2O	-	861 µl	Up 50 ml

LiCl wash buffer

**Final**	**Stock**	**Per ml**	**Per 50 ml**
0.25 M LiCl	8 M	32 µl	1.6 ml
Igepal 1%	100%	10 µl	500 µl
1% deoxycholate Na	-	10 mg	500 mg
1 mM EDTA	0.5 M	2 µl	100 µl
10 mM Tris pH8.1	1 M	10 µl	500 µl
Milli Q H_2O	-	946 µl	Up to 50 ml

TE

**Final**	**Stock**	**Per ml**	**Per 50 ml**
10 mM Tris pH8.1	1 M	10 µl	500 µl
1 mM EDTA	0.5 M	2 µl	100 µl
Milli Q H_2O	-	988 µl	49.4 ml

Elution buffer

**Final**	**Stock**	**Per 10 ml**	**Per 20 ml**
0.1 M $NaHCO_3$	-	84.1 mg	168.2 mg
1% SDS	20%	500 µl	1ml (2 ml with 10% SDS)
Milli Q H_2O	-	9.5 ml	Up to 20 ml

ChIP primers used:

PP2AC F: gatggggttttaccgtgttg

PP2AC R: ccaaagcaggtggatgactt

PP2AC F non-enriched region: atctcagccccatgttgttc

PP2AC R non-enriched region: gctcttgtgcagtcacatcg

SUPPLEMENTARY REFERENCES

Andegeko, Y., Moyal, L., Mittelman, L., Tsarfaty, I., Shiloh, Y., and Rotman, G. (2001). Nuclear retention of atm at sites of dna double strand breaks. J Biol Chem *276*, 38224-38230.

Bindra, R.S., Gibson, S.L., Meng, A., Westermark, U., Jasin, M., Pierce, A.J., Bristow, R.G., Classon, M.K., and Glazer, P.M. (2005). Hypoxia-induced down-regulation of BRCA1 expression by E2Fs. Cancer Res *65*, 11597-11604.

Bottini, A., Berruti, A., Bersiga, A., Brizzi, M.P., Brunelli, A., Gorzegno, G., DiMarco, B., Aguggini, S., Bolsi, G., Cirillo, F.*, et al.* (2000). p53 but not bcl-2 immunostaining is predictive of poor clinical complete response to primary chemotherapy in breast cancer patients. Clin Cancer Res *6*, 2751-2758.

Hammond, E.M., Denko, N.C., Dorie, M.J., Abraham, R.T., and Giaccia, A.J. (2002). Hypoxia links ATR and p53 through replication arrest. Mol Cell Biol *22*, 1834-1843.

Onder, T.T., Kara, N., Cherry, A., Sinha, A.U., Zhu, N., Bernt, K.M., Cahan, P., Marcarci, B.O., Unternaehrer, J., Gupta, P.B.*, et al.* (2012). Chromatin-modifying enzymes as modulators of reprogramming. Nature *483*, 598-602.

Palmero, I., and Serrano, M. (2001). Induction of senescence by oncogenic Ras. Methods Enzymol *333*, 247-256.

Parsons, J.L., Khoronenkova, S.V., Dianova, II, Ternette, N., Kessler, B.M., Datta, P.K., and Dianov, G.L. (2012). Phosphorylation of PNKP by ATM prevents its proteasomal degradation and enhances resistance to oxidative stress. Nucleic Acids Res *40*, 11404-11415.

Peters, A.H., O'Carroll, D., Scherthan, H., Mechtler, K., Sauer, S., Schofer, C., Weipoltshammer, K., Pagani, M., Lachner, M., Kohlmaier, A.*, et al.* (2001). Loss of the Suv39h histone methyltransferases impairs mammalian heterochromatin and genome stability. Cell *107*, 323-337.

Sirbu, B.M., Couch, F.B., and Cortez, D. (2012). Monitoring the spatiotemporal dynamics of proteins at replication forks and in assembled chromatin using isolation of proteins on nascent DNA. Nat Protoc *7*, 594-605.

Tanaka, M., Lai, J.S., and Herr, W. (1992). Promoter-selective activation domains in Oct-1 and Oct-2 direct differential activation of an snRNA and mRNA promoter. Cell *68*, 755-767.

Wilsker, D., Petermann, E., Helleday, T., and Bunz, F. (2008). Essential function of Chk1 can be uncoupled from DNA damage checkpoint and replication control. Proc Natl Acad Sci U S A *105*, 20752-20757.
